# Leiomyosarcoma of the portal vein: a case report and review of the literature

**DOI:** 10.1259/bjrcr.20160125

**Published:** 2016-12-23

**Authors:** Wan Hang Keith Chiu, Anthony WI Lo, Joseph KT Lee

**Affiliations:** ^1^Department of Diagnostic Radiology, The University of Hong Kong, Pokfulam, Hong Kong; ^2^Anatomical Pathology Division, Queen Mary Hospital, Pokfulam, Hong Kong; ^3^Department of Radiology, UNC School of Medicine, Chapel Hill, NC, USA

## Abstract

Leiomyosarcomas (LMS) are rare malignant tumours of smooth muscle origin predominately affecting females in their sixth decade. Only 2% of LMS arise from blood vessels and most are in the inferior vena cava. We present the first reported case of LMS of the portal vein in a male patient. Multidetector CT showed a large mass in the main portal vein, which was initially misinterpreted as a pancreatic cancer. Careful examination of the multidetector CT images showed radiological features of an intraluminal mass, and a preoperative diagnosis of primary LMS of the main portal vein was made. The patient underwent curative surgery and made an uneventful recovery. Awareness of this entity and recognition of the salient CT features may facilitate radiologists in making the correct preoperative diagnosis.

## Background

Leiomyosarcomas (LMS) are rare malignant tumours of smooth muscle origin, accounting for only 5–7% of soft tissue sarcomas. They predominantly affect females in the sixth decade of life and generally occur in the abdomen or the limbs. Only around 2% of LMS arise from the smooth muscle of the vessel wall, and the most common site is the inferior vena cava (IVC).^1,2^

LMS of the portal vein is extremely rare with four previous reports in the English literature, all of which were in females.^3–6^ In this article, we present the first reported case of LMS of the portal vein in a male patient with special emphasis on imaging findings seen on multidetector CT (MDCT) and positron emission tomography followed by a brief review of the existing literature.

## Case report

A 67-year-old male initially underwent contrast-enhanced multidetector CT of the abdomen in Sept 2014 at another hospital, which showed an incidental 4.2 × 3.9 cm mass in the upper abdomen. This was interpreted as a head of pancreas mass causing portal vein compression. The patient was asymptomatic; physical and laboratory examinations were all unremarkable.

The patient was lost to follow-up and did not receive any further investigation or treatment. Although still asymptomatic, he re-presented 12 months later for a repeat MDCT, which showed that the mass had increased in size to 4.8 × 5.0 cm. Cavernous transformation of the portal vein was also observed. The patient underwent exploratory laparotomy, which showed a tumour at the upper border of the pancreas and duodenum compressing the portal vein. A 1-cm lesion in the subcapsular aspect of segment II of the liver was also seen. The presumed pancreatic tumour was deemed unresectable and multiple biopsies were taken. The liver lesion was resected and the histology of both samples showed spindle cell tumour. The patient also underwent a positron emission tomography-CT, which showed the lesion to be hypermetabolic with SUVmax of 7.3. No metastatic disease was identified.

**Figure 1.**
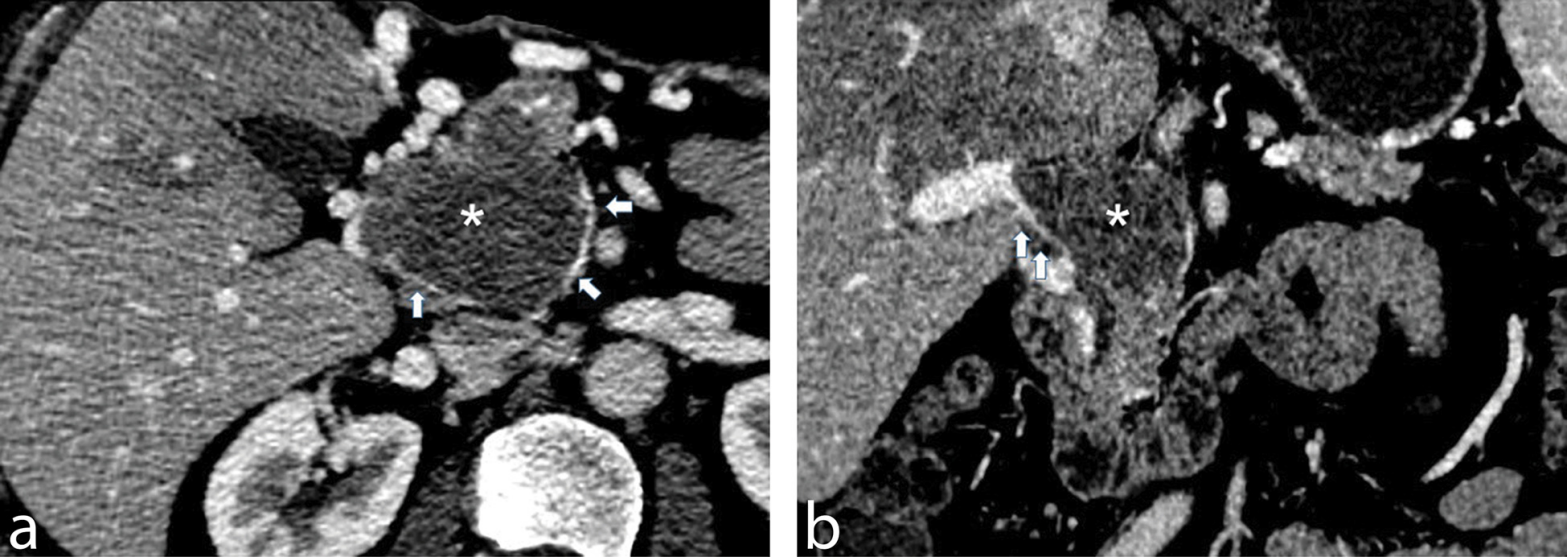
Multiplanar contrast-enhanced multidetector CT images reveal a mass in the portal vein (*). Axial CT image (a) shows numerous collaterals around the heterogeneously enhancing mass (*). There is a rim of contrast around the mass (white arrowheads) indicating that the mass has an intraluminal origin rather than tumour invasion from adjacent structures. Corresponding coronal CT image (b) shows a sharp interface with the appearance of a “beak” (white arrowheads) between the opacified main portal vein and the mass (*).

The patient was then referred to the hepatobiliary team in Queen Mary Hospital in Hong Kong for further assessment. The patient remained asymptomatic clinically with normal laboratory results including liver biochemistry (bilirubin 4 μmol l^–1^, alkaline phosphatase 54 U l^–1^, alanine aminotransferase 29 U l^–1^ and aspartate aminotransferase 23 U l^–1^). A repeat MDCT 2 months later showed that the lesion had a soft tissue density on non-contrast scans. The mass now measured 5.3 × 5.9 × 6.4 cm, extending from the superior mesenteric vein/splenic vein confluence to the porta hepatis. It exhibited heterogeneous enhancement with feeding vessels seen on the arterial phase, both within and around the mass. On the portal venous phase, numerous collaterals were seen surrounding the mass with a sharp interface between the mass and opacified portion of the main portal vein at the porta hepatis giving the appearance of a “beak” (Figure 1). The pancreatic duct was mildly dilated and measures 3 mm. The biliary tree was not dilated. There was splenomegaly measuring 13.5 cm craniocaudally. No gastroesophageal varices or ascites was appreciated. Based on these MDCT and histological findings, a preoperative diagnosis of primary leiomyosarcoma of the main portal vein was made.

**Figure 2.**
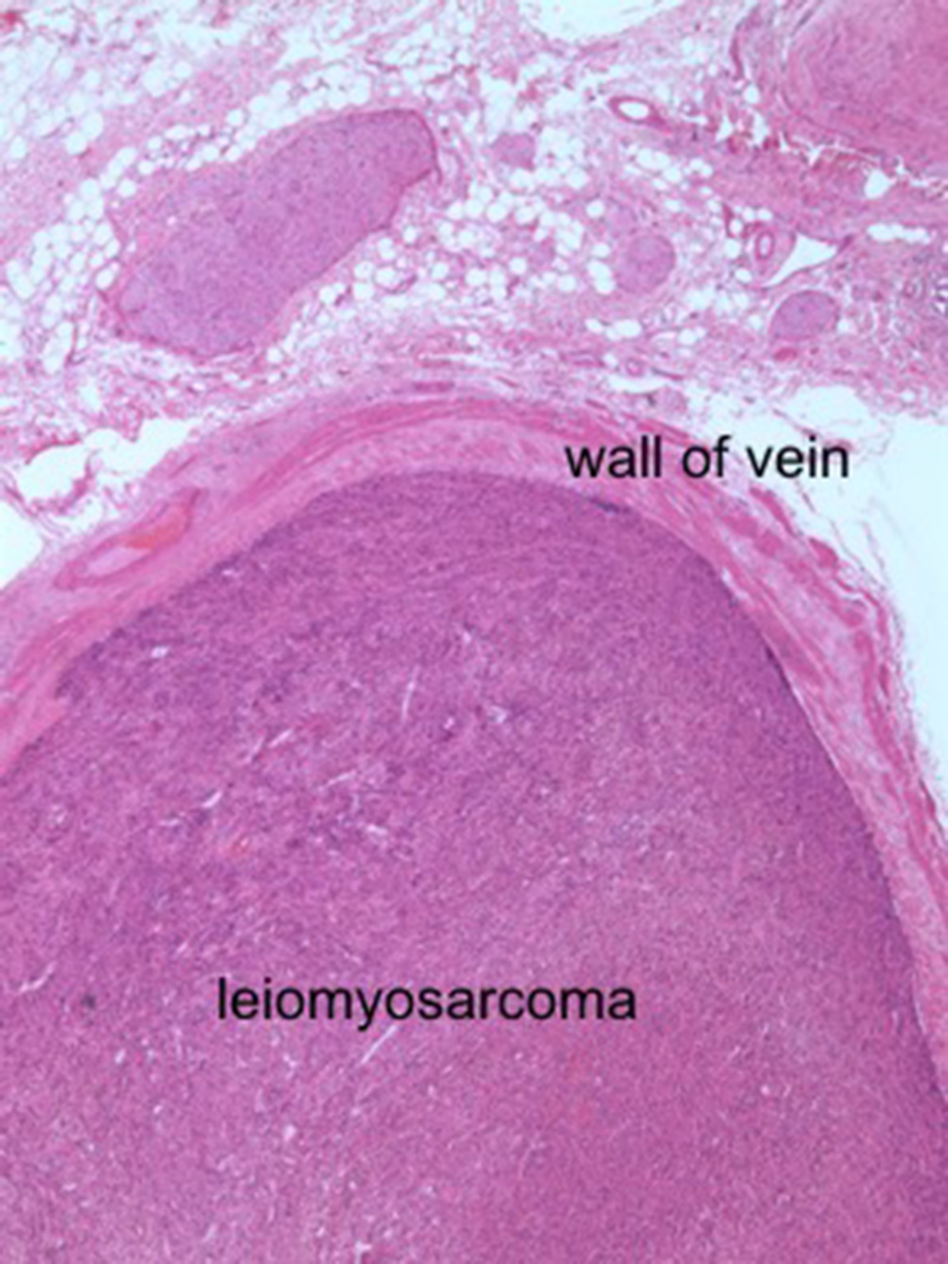
Microscopic findings of the resected specimen showing the leiomyosarcoma arising from within the portal vein and confined in the lumen (hematoxylin-eosin, 200× magnification).

The patient underwent Whipple’s procedure and portal vein reconstruction using cadaveric graft. Intraoperative findings confirmed the tumour arising from inside the portal vein and confined to the lumen with no macroscopic evidence of disease spread. Histological examination of the tumour showed spindle cells with enlarged, pleomorphic, hyperchromatic nuclei and abundant eosinophilic cytoplasm. Patchy coagulative necrosis was also seen. Mitotic figures were at 8 per 50 high power fields, and atypical mitotic figures were found. There was focal infiltration into adjacent pancreatic parenchyma. Immunohistochemical stains showed the tumour was positive for actin, h-caldesmon and desmin but negative for myogenin, c-kit and S100 protein. The final histological diagnosis was of a primary LMS of the portal vein (Figure 2). The patient made an uneventful recovery and no adjuvant radiotherapy or chemotherapy was given. He was followed up for 4 months in our institute with no complications.

## Discussion

Differential diagnoses of a portovenous mass could be “bland thrombus” or tumour thrombus from malignant aetiology including pancreatic adenocarcinoma, hilar cholangiocarcinoma and hepatocellular carcinoma. Distinguishing the two types of thrombus is essential as it significantly affects treatment options and prognosis. As in our case, enlargement of the PV and demonstration of feeding vessels within the thrombus indicate the presence of tumour thrombus rather than bland thrombus.^7^ Given the close proximity of the PV to pancreas, it is understandable that the lesion was initially misinterpreted as a primary pancreatic lesion causing portal vein thrombosis. In retrospect, the lack of concurrent dilated common bile and pancreatic ducts should make this diagnosis less likely.^8^ As for hepatocellular carcinoma and cholangiocarcinoma, tumour thrombus is often caused by direct invasion of the portal vein.^9,10^ Without a visible lesion in the adjacent liver parenchyma or in the hepatobiliary tract, these two differential diagnoses are also less likely. The identification of a rim of contrast around the lesion and “beak” appearance at the interface between the opacified portion of the PV and the mass further support the intraluminal nature in this case. While increased fludeoxyglucose uptake suggested malignant aetiology of the lesion, the presence of cavernous transformation of the portal vein and portal hypertension points to chronicity of the portal vein occlusion, a feature of a less aggressive lesion such as LMS. These patients are often asymptomatic although they can cause constitutional symptoms or non-specific abdominal pain.^11^ The slow-growing rate of this tumour results in insidious development of significant venous obstruction by allowing time for development of collateral circulation.^12^ Based on the radiological features, a primary PV neoplasm is the most likely diagnosis and LMS is the most common vascular neoplasm. The available histology further adds credence to this diagnosis while the resected liver lesion is considered a solitary metastasis.

LMS is divided into three major groups based on the origin of the tumour: soft tissue, cutaneous and vascular.^13^ LMS involving the vasculature is the least common and around 50% of them arise from the IVC. To date, approximately 300 cases have been reported in the literature.^14^ LMS in the PV is extremely rare and to the authors’ knowledge, there are only four previous reports of LMS in the PV, all of which were in females^3–6^ LMS involving other parts of the portovenous system are also rare and the published case reports in English are summarized in Table 1.

**Table 1.** Demographics, clinical characteristics, management and outcome of previous case reports of leiomyosarcoma of the portovenous system

Author	Age/Sex	Location	Tumour size	Presentation	Treatment	Outcome
Wilson^6^ & Madariaga^4^	28 F	PV	3.0 × 4.7 × 2.7 cm	Abdominal pain	Mesocaval shunt, TR, VR, RT	Alive at 6 years FU
Sundaresan^5^	67 F	Intrahepatic Left PV	30 × 15 × 10 cm	Epigastric pain	Hepatectomy, TR	Not reported
Boudjema^3^	44 F	PV	3 cm	Jaundice, pruritus, anorexia	cephalic duodeno-pancreatectomy, TR, VR	Recurrence at 27 months after surgery; died 20 months after recurrence
Our patient	67 M	PV	5.3 × 5.9 × 6.4 cm	Incidental	Liver wedge resection, Whipple procedure, TR and VR	Alive at 6 months FU
Goldin^17^	40 F	SMV	6 × 5.3 × 8 cm	Epigastric pain	Hepatectomy, TR, VR and RT	Alive at 12 months FU
Celdran^18^	60 M	SMV	4 cm	Epigastric pain	Hemicolectomy, TR and VR	Alive at 6 months FU
Leporrier^19^	50 M	SMV	3 cm	Epigastric pain	Whipple procedure, TR and VR	Alive at 18 months FU
Kumar^20^	62 F	SMV	13 × 10 × 7 cm	Incidental	Hemicolectomy, hepatectomy, TR, VR and CT	Alive at 33 months FU
Clemente^21^	66 F	IMV	--	Lower abdominal pain	Left colon resection, TR	Alive at 24 months FU
Cimino^22^	64 M	IMV	10 cm	General discomfort	Liver wedge resection, jejunal resection and TR	Alive with metastases at 13 months FU
Rodl^23^	67 M	SV	15 × 6 × 5 cm	Epigastric pain	Distal spleno-pancreatectomy	Alive at 36 months FU
Niver^24^ & Gage^25^	58 F	SV	3.5 × 3 × 3 cm	Epigastric pain	Distal spleno-pancreatectomy, VR	Alive at 15 months FU
Aguilar^26^	66 F	SV	12 × 9 × 6 cm	Epigastric pain	Distal spleno-pancreatectomy, CT	Alive at 12 months FU
Patrono^27^	58 F	SV	1.5 cm	Epigastric pain	Local excision, end-to-end splenic vein anastomosis	Alive at 12 months FU

PV, portal vein; SMV, superior mesenteric vein; IMV, inferior mesenteric vein; SV, splenic vein; TR, tumour resection; VR, venous reconstruction; RT, radiotherapy; CT, chemotherapy; FU, Follow up; F, Female; M, Male.

The classical appearance of LMS on MDCT is a circumscribed soft tissue mass, often with necrosis, cystic degeneration and haemorrhage.^15,16^ This contrasts with the radiological description of LMS of the PV. Wilson^6^ described a lesion filling and expanding the lumen of the PV on ultrasound imaging. Madariaga^4^ confirmed the intraluminal nature of the lesion in surgery. Sundaresan^5^ described a nodular vascular tumour within the left lobe of the liver and three smaller satellite lesions in the right lobe, all of which had enhancement characteristics of haemangioma on CT. Histological examination showed a vein with pleomorphic cells streaming out from the media. Boudjema^3^ showed an enhancing lesion adjacent to the PV compressing the common bile duct. The tumour was found on the PV intraoperatively. The varied appearances correlate with different degrees of extravascular components of the tumours and the appearance in the current case reflects the completely intraluminal nature of the tumour, similar to those described by Wilson and Madariaga.^4,6^

As in other LMS, surgical resection with or without venous reconstruction is the treatment of choice for this group of patients. Adjuvant chemotherapy and radiotherapy were used in some cases although the evidence of the efficacy of these treatments is limited owing to the rarity of these cases. In general, for LMS in the IVC, the overall 5-year survival rate is 49% with up to 50% recurrence within 30 months. For non-IVC LMS, the 4-year survival rate is 32%.^2,11^ The resection margin, tumour size and degree of tumour differentiation are thought to be important factors to overall survival.^15^ In our case, the patient underwent surgery without adjuvant treatment as the tumour was deemed to be of low grade.

## Learning points

1. LMS of the PV is rare. Surgical resection is currently the only realistic chance of cure and long-term surveillance after resection is recommended given the high potential for recurrence and metastatic disease. Awareness of this entity and recognition of the salient CT features as described may facilitate radiologists in making the correct preoperative diagnosis.

## Consent

Written informed consent for the case to be published (including images, case history and data) was obtained from the patient(s) for publication of this case report, including accompanying images.
